# Motivating actions to mitigate plastic pollution

**DOI:** 10.1038/s41467-019-12666-9

**Published:** 2019-10-08

**Authors:** Lili Jia, Steve Evans, Sander van der Linden

**Affiliations:** 1 Department of Engineering, University of Cambridge, Cambridge, CB3 0FS UK; 2 Department of Psychology, University of Cambridge, Cambridge, CB2 3EB UK

**Keywords:** Psychology and behaviour, Sustainability, Decision making, Society

## Abstract

Designing effective policy interventions to motivate mitigation actions requires more realistic assumptions about human decision-making based on empirical evidence from the behavioural sciences. We therefore need to consider behavioural rather than only economic costs and benefits in policy intervention designs.

## Abstract (web summary)

Plastic pollution is a purely anthropogenic problem and cannot be solved without large-scale human action. Motivating mitigation actions requires more realistic assumptions about human decision-making based on empirical evidence from the behavioural sciences enabling the design of more effective interventions.

Reducing plastic pollution has been pushed to the top of the global policy agenda. As of 2015, 6.3 billion tonnes of plastic waste has been generated, 79% of which has ended up in landfills or the natural environment^1^. This huge amount of plastic pollution is threatening our marine, freshwater, and soil environment and potentially the safety of our entire food system^1^. Accordingly, a number of governments have taken initiatives against plastic pollution: Canada aims to ban single-use plastics by 2021^2^; Peru banned single-use plastics in 76 natural and cultural protected areas^2^; and 170 United Nations Member States pledged to reduce the use of plastics “significantly” by 2030^3^.

While plastics are ubiquitous in daily life, reducing plastic pollution requires actions from all stakeholders to encourage a more sustainable use of plastics. For example, individuals need to reduce consumption and minimise their plastic waste^4,5^. Industries need to take on producer responsibility^4,5^ and design plastic products that minimise negative environmental impacts throughout their lifecycles^4–6^. Governments need to raise public awareness about plastic waste, restrict toxic^6^ and avoidable plastic usages^6^ and improve plastic waste management for recycling and remanufacturing^4–6^. Many of these actions, however, are currently lacking. From 2009 to 2017, global demand for plastics increased at 4% annually and plastic waste has continued to grow^7^. Industries rarely explore the potential of eco-design to reduce, reuse and recycle plastic waste^4^—most of which has gone to incineration or landfills leading to a huge loss in natural resource value^8^.

## Beyond Homo Economicus

**Fig. 1:**
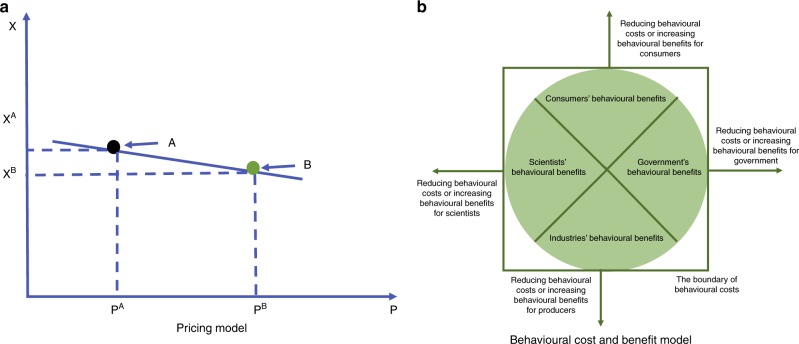
The graph on the left **a** shows the pricing model in neoclassical economic theory where the quantity demanded of a normal good (X) has a negative relationship with its price (P). If X’s price increases from P^A^ to P^B^, the quantity demanded of X will decrease from X^A^ to X^B^. The diagram on the right **b** shows how behavioural costs and benefits may affect demand. The green area in the circle indicates the behavioural benefits of reducing plastic pollution for consumers, government, industries, and scientists, respectively. The square represents the constraints of behavioural costs. The arrows outside of the square indicate that actions (to mitigate plastic pollution) can be motivated either by increasing behavioural benefits or by decreasing the behavioural costs of the stakeholders

Designing policy to motivate mitigation actions requires a better understanding of behavioural theory. Policy-makers often rely on an outdated mental model of human behaviour^9^ where it is assumed that price policy alone can successfully motivate mitigation actions. For example, taxing plastic products is supposed to increase its price and accordingly, decrease its consumption^9^. It is derived from neoclassical economic theory: where demand for the quantity of a normal good (any good for which demand increases with increased income) has a negative relationship with its price (Fig. 1a)^9^. Because the economic costs of many plastic products are often small, the impact of a change in price on plastic demand reduction is likely to be too modest to achieve a significant societal reduction in plastic pollution. Thus neoclassical economic theory oversimplifies human behaviour by solely relying on economic incentives^10^ and loses its predictive power when more behavioural incentives are involved, e.g., time, effort, and convenience. To design more effective policy interventions, it is therefore critical to improve our theoretical understanding of behavioural incentives which go above and beyond mere economic incentives^11–13^.

We suggest that behavioural theory is a more promising candidate for helping policy makers motivate plastic mitigation actions. Behavioural theory tells us that an individual makes a demand decision by weighing behavioural costs and benefits derived from values in consumption activities^11–13^. Behavioural costs include but are not limited to money, availability, time, effort and distance^11–13^. An individual’s mitigation actions can be motivated through increasing behavioural costs, such as bans and taxation. For example, estimates reveal that banning and taxing single-use plastic bags in European countries have motivated consumers to reduce their plastic bag waste by 66–90%^14^. Accordingly, it is often believed that increasing behavioural costs is the most effective way to motivate mitigation actions. Its application, however, is limited to avoidable plastic wastes, such as straws^2^, and the approach faces difficulty to help achieve a more general reduction of plastic consumption. For example, behavioural research on the single-use carrier bag charge in Wales finds that although the policy increased bag reuse, such “external” incentives do little to motivate wider sustainable views and behaviours^15^. More effective policy interventions are therefore needed to motivate consumers to curb the consumption of plastics.

## Behavioural costs and benefits

Behavioural benefits provide an alternative pathway to motivating mitigation actions (Fig. 1b). These benefits can be psychological in nature and extend far beyond mere economic value. Behavioural benefits are broadly known to be closely associated with an individual’s personal goals which are categorised into three groups:^13^ (1) hedonic goals (e.g., avoiding effort, gaining pleasure); (2) gain goals (e.g., money and social status); and (3) normative goals (doing what is right, normative, or appropriate). Mitigation actions are more likely to be motivated when they consistently deliver behavioural benefits to multiple or all three goals^13^. For example, recycling plastic waste can give rise to behavioural benefits by eliciting positive feelings—also known as environmental ‘warm-glow’—which can be leveraged to motivate behavioural mitigation actions^16^.

Gaining a better understanding of behavioural costs and benefits will lead to more effective policy-making to motivate plastic pollution mitigation. To illustrate, consider that while conscious actions require more cognitive involvement (implying higher behavioural costs), subconscious or intuitive actions require little to no cognitive effort and thus have lower associated behavioural costs^13^. A plastic pollution mitigation action is more likely to be motivated if it requires less cognitive effort (a clear behavioural benefit). For example, in some European countries, container refund machines are placed at supermarkets to make it easier for consumers to recycle their plastic bottles when they go grocery shopping. This indicates that industries can help their customers reduce plastic pollution more easily when they improve their products and service designs^5^.

It is often assumed that industries have little incentive to take actions to reduce plastic pollution because of the fear that it may reduce their profits. Yet, consumers are often more concerned with the functionality delivered by plastic products rather than plastics themselves^10^. This means that industries can reduce plastic waste and production cost without sacrificing the quality of their services. For example, simply providing a refillable option for personal and home-care products could help households save about 5% of plastic packaging materials^5^. To underline this point, it is estimated that 20% of plastic packing can be saved globally through reuse and the cost savings on those materials amount to about $9 billion^5^.

Thus, even though the use of plastics is sometimes unavoidable, industries can mitigate plastic pollution through advancing the design of their products^5^. Estimates indicate that 50% of plastic packaging can be recycled through improving the design of packaging. For example, using PET to replace PVC, PS, and EPS plastics can remove the source of contamination in the recycling process^6^. If industries choose clear or light-coloured rather than coloured or opaque materials in product design, it will increase the quality of recycled plastics^6^. Despite these mitigation potentials in product design, industries often lack incentives to explore it^5,6^.

## Designing better policies: towards a common goal

Effective policies should therefore motivate industries and individuals to reduce plastic pollution collaboratively. The Extended Producer Responsibility (ERS) scheme in the EU has increased the recycling rate of plastic packaging^5^, however, it fails to encourage industries to explore the full mitigation potential of plastic pollution reduction^6^. On one hand, the low compliance fees for plastic producers do not provide sufficient incentive for producers to adopt an eco-design. On the other hand, the fragmentation of collection and sorting systems^6^ and the incoherent regulation of ERS among different EU member states create barriers for producers to take more responsible actions to reduce plastic pollution^5^. These behavioural barriers need to be considered in plastic reduction policy design, for example, extending ERS to more plastic products, increasing the ERS compliance fee for producers^5^, harmonising ERS regulation^5,6^ and integrating higher environmental standards into the standards of plastic products^6^.

The actions of individuals, industries and policy makers are not independent from one another. For example, when government policy raises awareness of reducing plastic waste in daily life and industries provide convenient options for consumers to refill personal and home-care products^6^, it will motivate more consumers to reduce their plastic consumption. These coordinated actions, however, are rarely considered in the design of interventions. How to effectively integrate the knowledge of the behavioural sciences into the design of mitigation action interventions and motivate collaborative efforts across stakeholders remains a major policy challenge. Both basic and applied research is urgently needed to help design more effective interventions and motivate mitigation actions at population level.

Mitigating plastic pollution is a global challenge. Although scientists, policy makers, industries and consumers have started to respond to this challenge, their actions are often motivated solely by considering behavioural costs. Banning single-use plastics may not always provide an effective solution for all types of plastic use in daily life. Increasing behavioural benefits and lowering the behavioural costs of reducing plastic pollution for decision-makers offers a more promising pathway to large-scale societal mitigation actions. This requires deepening our theoretical and empirical understanding of behavioural incentives and making more behaviourally realistic assumptions in designing policy interventions. It cannot be achieved by behavioural scientists alone, however, and will require a collaborative effort from all stakeholders, including behavioural scientists, engineers, chemists, ecologists, policy makers, industries, and consumers. Together we can unlock the behavioural benefits of mitigating plastic reduction and move toward a society free from plastic pollution.
